# Travel Burden as a Measure of Healthcare Access and the Impact of Telehealth within the Veterans Health Administration

**DOI:** 10.1007/s11606-023-08125-3

**Published:** 2023-06-20

**Authors:** Zachary Hahn, John Hotchkiss, Charles Atwood, Connor Smith, Annette Totten, Eilis Boudreau, Robert Folmer, Priyanka Chilakamarri, Mary Whooley, Kathleen Sarmiento

**Affiliations:** 1 Togus VA Medical Center, 1 VA Ctr, Augusta, ME 04330 USA; 2 Pittsburgh VA Medical Center, Pittsburgh, PA USA; 3 Portland VA Medical Center, Portland, OR USA; 4 Oregon Health and Science University, Portland, OR USA; 5 San Francisco VA Medical Center, San Francisco, CA USA

**Keywords:** Travel distance, Travel burden, Healthcare access, Telehealth, Sleep medicine

## Abstract

**Background:**

Travel is a major barrier to healthcare access for Veteran Affairs (VA) patients, and disproportionately affects rural Veterans (approximately one quarter of Veterans). The CHOICE/MISSION acts’ intent is to increase timeliness of care and decrease travel, although not clearly demonstrated. The impact on outcomes remains unclear. Increased community care increases VA costs and increases care fragmentation. Retaining Veterans within the VA is a high priority, and reduction of travel burdens will help achieve this goal. Sleep medicine is presented as a use case to quantify travel related barriers.

**Objective:**

The Observed and Excess Travel Distances are proposed as two measures of healthcare access, allowing for quantification of healthcare delivery related to travel burden. A telehealth initiative that reduced travel burden is presented.

**Design:**

Retrospective, observational, utilizing administrative data.

**Subjects:**

VA patients with sleep related care between 2017 and 2021. In-person encounters: Office visits and polysomnograms; telehealth encounters: virtual visits and home sleep apnea tests (HSAT).

**Main Measures:**

Observed distance: distance between Veteran’s home and treating VA facility. Excess distance: difference between where Veteran received care and nearest VA facility offering the service of interest. Avoided distance: distance between Veteran’s home and nearest VA facility offering in-person equivalent of telehealth service.

**Key Results:**

In-person encounters peaked between 2018 and 2019, and have down trended since, while telehealth encounters have increased. During the 5-year period, Veterans traveled an excess 14.1 million miles, while 10.9 million miles of travel were avoided due to telehealth encounters, and 48.4 million miles were avoided due to HSAT devices.

**Conclusions:**

Veterans often experience a substantial travel burden when seeking medical care. Observed and excess travel distances are valuable measures to quantify this major healthcare access barrier. These measures allow for assessment of novel healthcare approaches to improve Veteran healthcare access and identify specific regions that may benefit from additional resources.

**Supplementary Information:**

The online version contains supplementary material available at 10.1007/s11606-023-08125-3.

## INTRODUCTION

Veterans experience many barriers to healthcare access. Travel- and transportation-related barriers have been shown to be the greatest challenges in healthcare access^1–3^. These barriers are compounded for rural Veterans due to longer travel distances, limited transportation options, and greater financial constraints associated with travel.^1–3^ Approximately one quarter of US Veterans live in rural areas, tend to be older, and have worse health, lower socioeconomic status, and fewer community resources^4,5^.

Veterans now have increased access to community care (care outside the VA but paid by the VA) with the passage of the CHOICE/MISSION acts. Excessive barriers to VA care decrease the utilization of VA exclusive care^6^, and the number of Veterans seeking community care has been increasing^7^. Rural Veterans have limited access to specialty care, increasing dependence on community care. The threshold to seek community care due to excessive access barriers has likely shifted with increased community care access. While increased healthcare access for Veterans is desirable, the effects of increased community care on Veteran outcomes remain unclear. Proposed benefits of community care include increased timeliness and decreased travel distance; however, there is increased fragmentation and cost to the VA.

Compared to the general population, VA enrollees tend to be older and have more complex medical problems^8^. Despite a greater burden of disease, “the VA often (but not always) performs better than or similarly to other systems of care with regard to the safety and effectiveness of care.^9^” Increased access to community care has likely decreased continuity of care, which is detrimental to Veterans. Continuity of care has been widely shown to reduce mortality, morbidity, admission rates, re-admission rates, and emergency department utilization and improve management of chronic disease^10–16^.

Retaining Veterans within the VA improves care coordination and decreases fragmentation, both associated with improved outcomes, and thus an important goal. To improve the VA healthcare system and reduce barriers to access, especially for rural Veterans, we must first quantify those barriers. By reducing access barriers, we can increase exclusive VA care, and thereby improve Veteran continuity of care and health outcomes. Sleep medicine is presented as a use case to quantify travel distance and explore methods for improvement. Sleep-related conditions are common among Veterans^17–19^, with obstructive sleep apnea (OSA) affecting an estimated 24% of the VA population^20^. Additionally, the prevalence of sleep disorders has been increasing during the last decade^20–26^, and likely remain under-reported and under-diagnosed^27,28^. The population of patients requiring sleep care “greatly outstrips” the available resources, and patients often must travel long distances for care or experience long wait times^17^. The VA is not immune to this issue and has taken strides to improve access through a nationwide TeleSleep Program^21^.

### Objective

In this study, we present two measures of healthcare access: *Observed* and *Excess Travel Distances.* They relate to the concept of travel burden and may inform on different aspects of the healthcare system and access. Observed travel distance helps to quantify the Veteran’s experience while seeking healthcare. Excess travel distance helps to elucidate inefficiencies in the healthcare system by identifying when and where Veterans traveled farther than necessary to receive VA care. We explore how these measures could be utilized to characterize the efficiency of healthcare delivery to facilitate evidence-based system improvement. The effects of a telehealth initiative to reduce travel by comparing in-person and telehealth equivalents are presented.

### Travel Burden: A Common Barrier to Healthcare Access

Travel burden is a complex and poorly defined concept that can have a significant impact on healthcare access and may disproportionately affect Veteran populations based on age, rurality, and economic status, among other factors. The Veteran’s travel burden may depend on healthcare facility distance, geography, demographics, socioeconomics, weather/season, road conditions, and comorbidities. Excessive travel can inhibit Veterans from seeking care, delay follow-up, increase “no show” rates, and increase the risk of motor vehicle accidents. Many of the concepts surrounding travel burden are difficult to quantify and a gold standard definition is lacking. Travel distance is a component of travel burden, but does not wholly encompass the concept, and its impact varies between individuals. Despite these limitations, travel distance provides an objective measure that can inform at the system level.

### Potential Solutions—Telehealth Reductions in Travel Burden

The VA has established an Enterprise-wide TeleSleep initiative with the specific aim of increasing sleep care access for rural Veterans^21^. Adequate treatment of OSA leads to a significant improvement in Veteran’s quality of life, including PTSD symptoms^29–32^. Telehealth technologies decrease wait times^33^, are efficacious in the management of sleep disorders, and are well received by most Veterans^34–41^. Our prior work quantified the potential reduction of travel achievable through widespread TeleSleep use^42^. One small study previously examined the impact on travel for Veterans using telehealth^43^. Here we demonstrate the impact at the national level to allow for evaluation of how telehealth programs can remove a major barrier to healthcare access for Veterans, and especially those in rural areas.

## METHODS

### Data Sources

All work was performed as part of VA quality improvement operational analyses and did not require waiver of informed consent or IRB review. All analyses were performed in R version 4.1.2 and RStudio within the VA Informatics and Computing Infrastructure virtual computer network. Retrospective administrative data were obtained from the VA Corporate Data Warehouse (CDW)^44,45^, the repository for all VA clinical data, specifically the outpatient workload table. Only VA encounters are considered here; however, the following measures remain valid if community care data are incorporated.

The data points obtained from the CDW for each clinical encounter include the following: Unique Veteran identifier, fiscal year (FY), VA facility and geocoordinates, encounter type, and Veteran’s primary residence geocoordinates. Geocoordinates refer to latitude and longitude. These values are typically reverse geocoded from a street address, but when this level of precision is unavailable, the geocoordinate of the zip code centroid was utilized. The CDW contains accurate geocoding information for each Veteran, but similar results can be obtained based on commonly available location information (e.g., city, county, or zip code).

Veteran encounters in FY2017-2021 were examined for relevant clinical encounter types. Encounter types were based on recorded Stop Codes and Current Procedural Terminology (CPT) codes. The specific breakdown of Stop Codes, CPT codes, and corresponding clinic type are outlined in Appendix 1. Veterans obtaining in-person sleep care were the target encounter types, which include provider office visits and polysomnograms (PSG). The telehealth equivalents of in-person care (virtual provider visit and HSAT, respectively) were utilized to quantify the reduction of travel distance afforded by TeleSleep. This analysis addressed sleep related care as a use case, but these methods are specialty agnostic and can be applied to other clinical encounters.

### Travel Burden: Observed and Excess Distances

Travel distance was defined as the round trip, City Block distance in miles between a Veteran’s primary residence, and the treating VA healthcare facility. An Approximated Euclidean Distance (AED) measure was used to measure the distance between the two points. Specifics on City Block distance and the calculation of the AED are included in Appendix 2.

Although more accurate measures of travel distance exist, studies comparing Euclidean distance, road network distance, and travel time have found a high degree of correlation across many geographic settings^46–48^. Surveys of rural VA enrollees have shown that travel distance is the greatest barrier to healthcare access^1^. City Block distance is calculated independent of the underlying road network and approximates true travel distance. It is computationally efficient, allowing for large network analysis. City Block distance is utilized rather than straight-line distance as it represents a more conservative measure of travel distance.

*Observed Travel Distance* is the distance between a Veteran’s primary residence and the VA facility where care was rendered. In our analysis, any observed distances over 400 miles are censored from further analysis, as this often represents Veterans who spend part of the year in different regions of the country (“snowbirds”). The VA healthcare facility where a Veteran received care, the *Observed Facility*, may not be the closest facility to their residence that offers the service. The *Nearest Facility* (*NF*) is the VA facility closest to the Veteran’s home that offers the medical service. The *Shortest Travel Distance* represents the distance a Veteran would have traveled to receive care at the NF. A *Distant Facility* (*DF*) is any VA facility where a Veteran received care that is not the NF.

*Excess Travel Distance* is the difference between the observed and shortest travel distances and occurs when a Veteran received care at a DF. The inclusion of community care hospitals would require the development of a nationwide list of services offered at each hospital to determine NF status. These measures could be expanded if such information were available.

Many patients live relatively equidistant between two facilities (20mi to one and 25mi to another). Thus, from a practical perspective, either could be considered acceptable; however, from a technical perspective, one will be closer than the other (20mi). A threshold distance of 40 excess round-trip miles was set to determine NF status (the facility 25mi away would have a 10mi excess round trip distance compared to the 20mi facility). Any excess distance below this threshold was ignored and the patient was assumed to have traveled to the nearest facility.

Observed and excess distance measures can be evaluated at the patient, facility, or geographic level. Total distances and median distances are reported at the patient level, for each clinic type, and at the county level. Facility-level analysis and network efficiency are considered, but detailed exploration of this topic is beyond the scope of this paper. Any reported station numbers/names are de-identified.

### Effects of Virtual Care on Travel Burden

*Avoided Travel Distance* is defined as the round trip, City Block distance between the Veteran’s primary residence and the nearest VA healthcare facility offering the in-person equivalent of the telehealth service. Community care hospitals are not considered. It was assumed that telehealth visits occurred in the Veteran’s home, and that HSAT devices were mailed to the Veteran, and thus Veterans incurred no travel burden for these encounters. Geocoordinates of the VA facility and Veteran’s primary residence were utilized to calculate avoided travel distances.

## RESULTS

### Care Utilizations Trends

**Figure 1:**
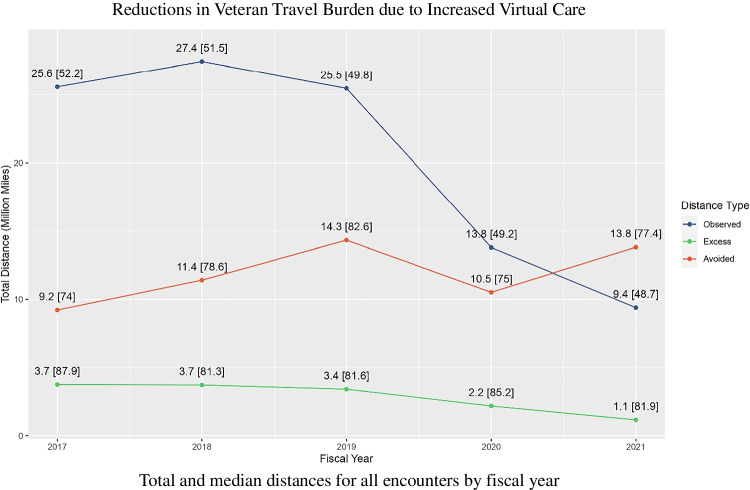
Total distances are presented in millions of miles, with the median patient distance in miles shown in brackets. The total observed travel distances associated with in-person care have been down trending, while the total avoided travel distances due to virtual care have been increasing. Median distances have remained relatively constant. In-person encounter types: Office visits and sleep lab studies (PSG). Virtual encounter types: Virtual provider visits and home sleep apnea tests (HSAT). Observed and excess distances refer to in-person encounters only. Observed distance represents the distance Veterans traveled to receive in-person care. Excess distance represents the extra distance Veterans traveled to receive care if they were not seen at the closest VA facility offering the service. Avoided distances refer to virtual encounters only and represent the distance Veterans would have traveled to receive the equivalent in-person care at the nearest VA facility offering the service. It is assumed virtual care occurs in the home and no travel burden is incurred.

A total of 1,253,980 in-person office visits (606,046 unique Veterans) and 505,373 TeleSleep encounters (359,434 unique Veterans) were included in the analysis (Office: 980,824; PSG: 273,156; Virtual: 73,308; HSAT: 432,065) for FY2017-2021. Table 1 provides clinic visit counts in thousands of visits and total distances in millions of miles for each clinic type by FY. The number of encounters for each clinic peaked between FY2018-2019 and have since down trended, except for HSATs which increased in FY2021. The observed and excess travel distances follow similar downward trends. Avoided travel distances have generally increased over this period, driven by increased utilization of HSAT devices. The values of observed travel distance and avoided travel distance crossed during FY2020 (Fig. 1).

**Table 1:** Shifting trends in sleep care and associated travel burden.

Fiscal year					
Clinic	2017	2018	2019	2020	2021
Office Visit count (K)	239.4	263.4	253.8	140.0	84.1
Total observed (M mi)	19.6	21.3	19.9	10.9	6.4
Median observed (mi)	50.9	50.2	48.7	48.6	47.8
Total excess (M mi)	3.1	3.0	2.8	1.9	0.8
Median excess (mi)	84.9	80.2	80.1	83.3	81.6
PSG Visit count (K)	66.1	67.8	64.5	35.7	39.1
Total observed (M mi)	6.0	6.2	5.6	2.9	3.0
Median observed (mi)	57.6	58.3	55.6	52.3	50.9
Total excess (M mi)	0.6	0.7	0.6	0.3	0.3
Median excess (mi)	110.7	97.7	94.6	94.1	92.8
Virtual Visit count (K)	14.1	17.2	22.1	12.3	7.6
Total avoided (M mi)	2.0	2.4	3.4	1.7	1.3
Median avoided (mi)	123.3	120.5	133.4	124.3	153.7
HSAT Visit count (K)	67.6	80.1	93.2	79.7	111.4
Total avoided (M mi)	7.2	9.0	10.9	8.8	12.5
Median avoided (mi)	67.0	71.5	72.9	69.3	74.0

Avoided travel distance now exceeds the observed travel distance due to increased HSAT utilization. The volume of most encounter types had been stable or up trending through FY2019. A sharp downturn followed in FY2020, except HSAT utilization, which has continued to increase. All visit counts are in thousands of patient visits per clinic type. Total distances are in millions of miles, and median distances are in miles. Observed distance represents the distance Veterans traveled to receive in-person care. Excess distance represents the extra distance Veterans traveled to receive care if they could not be seen at the closest VA facility offering the service. Avoided distances refer to virtual encounters only and represent the distance Veterans would have traveled to receive the equivalent in-person care

*PSG* polysomnography, *HSAT* home sleep apnea test

### Travel Distance Variation

**Figure 2:**
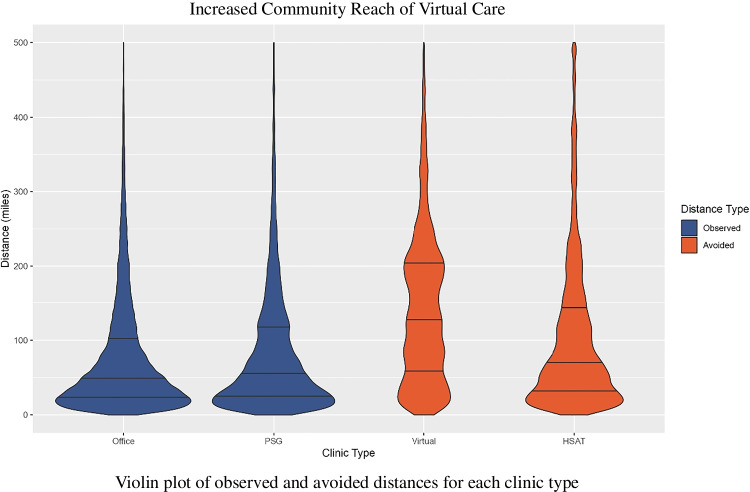
Distances are presented in miles. Most Veterans seeking in-person care have relatively short travel distances, while virtual care treats patients much further away from the VA facility. Graph width represents relative number of Veterans at the specified distance. In-person encounter types: Office visits and sleep lab studies (PSG). Virtual encounter types: Virtual provider visits and home sleep apnea tests (HSAT). Distances shown for in-person visits are the distance traveled by a Veteran to receive care. Distances shown for virtual visits are the distance a Veteran would have traveled to receive the equivalent in-person care. The 25th, 50th (median), and 75th quantiles are shown for each clinic type as horizontal lines within the violin plot. The limit of the graph is drawn at 500 miles for clarity, a small number of encounters do have distances beyond this point. PSG, polysomnography. HSAT, home sleep apnea test.

The distance distribution varies depending on clinic type. Figure 2 depicts the distance distribution in miles of each clinic type, with the 25th, 50th (median), and 75th quantiles represented by horizontal lines. For in-person visits, the distances represent observed travel distances, and for telehealth visits the distances represent avoided travel distances. The upper limit of the graph was set at 500mi to improve presentation, although the maximum distance for each clinic type is often much higher (Office: 800mi; PSG: 800mi; Virtual: 1192mi; HSAT: 3756mi) and account for 0.52% of office visits and 1.68% of TeleSleep visits.

### Vulnerability of Select Veterans to Excessive Travel

Table 2 depicts the median observed and excess distances in miles stratified by clinic type and care location (NF/DF). Again, NF patients received care at the nearest VA facility that offers the medical service, and thus did not incur excess travel. DF patients incur excess travel by receiving care at a VA facility farther from home, despite living closer to a VA facility that offers the service. Not surprisingly, the observed travel distances are larger for DF patients compared to NF patients. The excess component of their travel is nearly double that of the observed travel distance for NF counterparts. Across all years, the average percentage of NF utilization is 90.82% (Office: 90.05%; PSG: 93.58%).

**Table 2:** Vulnerability of select veterans to excessive travel distance.

Clinic type			
Care location		Office	PSG
All	Visits (K)	980.8	273.2
	Median observed (mi)	49.5	55.3
Nearest Facility	Visits (K)	883.2	255.7
	Median observed (mi)	43.4	50.4
Distant Facility	Visits (K)	97.6	17.5
	Median observed (mi)	192.2	245.1
	Median excess (mi)	81.9	99.1

Veterans who receive care at a distant facility face a significantly greater travel distance for in-person sleep care, with travel distance nearly 4 times their counterparts receiving care at the nearest facility. “Nearest facility” indicates the Veteran received care at the nearest VA facility offering the service. “Distant facility” indicates the Veteran did not receive care at the closest VA facility offering the service and incurred an excess travel burden

Distances listed are the median observed or excess travel distance in miles. Values are listed for all patients and stratified by care location (nearest facility vs distant facility). Only Veterans who receive care at a distant facility incur excess travel

*PSG* polysomnography

### Reduction of Travel Through TeleSleep

TeleSleep visits represent avoided travel. Figure 2 demonstrates that the median-avoided travel distance for TeleSleep encounters is greater than the observed travel distance for in-office counterparts (Office: 49.5mi vs Virtual: 128.2mi; PSG: 55.3mi vs HSAT: 71.2mi).

A total of 10.9 million miles of travel was avoided over the 5-year period through use of telehealth, and 48.4 million miles of travel was avoided using HSAT devices.

### Geospatial Analysis

**Figure 3:**
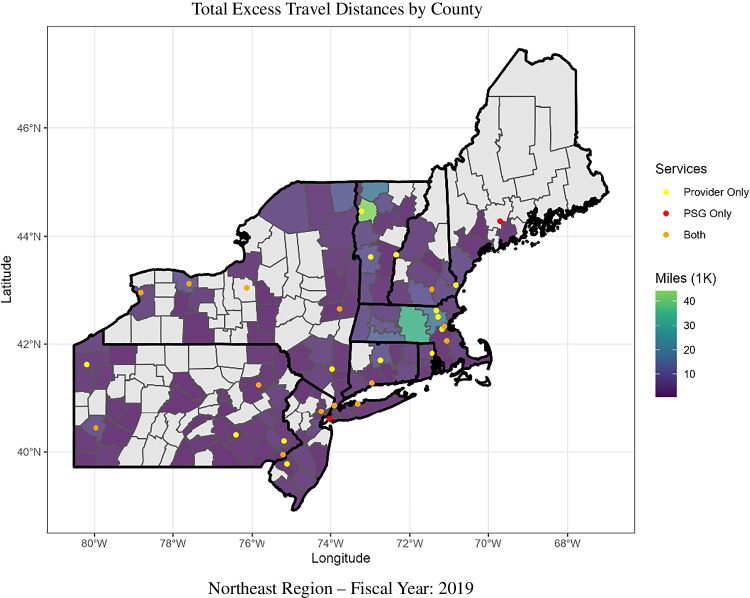
Total distances are presented in thousands of miles. Excess travel distance represents the difference in distance between where the Veteran received care (observed facility) and the facility closest to the Veteran’s home that offers the medical service of interest (optimal facility). When these two facilities are the same no excess travel distance in incurred, when they differ Veterans travel an excess distance to receive care. The total excess travel distance for all Veterans is shown aggregated at the county level. Only counties with a total excess travel distance of 5000 miles or greater are shown.

**Figure 4:**
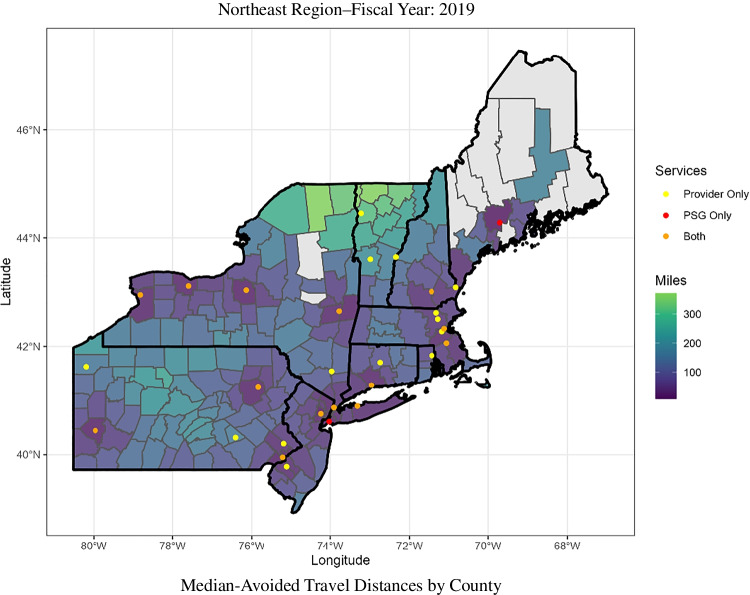
Median distances are presented in miles. Avoided travel distance represents the distance between the Veteran’s primary residence and the nearest VA healthcare facility offering the in-person equivalent of the telehealth service. It represents the distance a Veteran would have to travel to receive in-person care and was thus avoided by receiving virtual care. It is assumed that virtual care occurs in the home, and HSAT devices are mailed to the Veteran, and thus no travel burden in incurred for these encounters. The median-avoided travel distance for all Veterans is shown aggregated at the county level. Only counties that have a median avoided distance greater than zero are shown.

Figures 3 and 4 show two strategies for geographic aggregation of excess and avoided travel distances at the county level. Only the Northeast region is shown, but maps of other regions can be found in Appendix 3. Figure 3 displays the total excess travel distance for each county. Only counties with a total excess travel distance greater than 5000 miles are shown. Figure 4 displays the median-avoided travel distance by county. Only counties with a median-avoided distance greater than zero are shown.

## DISCUSSION

Veterans within the VA often experience a substantial travel burden when seeking sleep care. We propose two distance measures to better quantify the impact of travel on care access. While in-person visits have decreased, the median observed travel distance remained relatively constant. The observed travel distance can be viewed as a Veteran-level measure and is related to where the Veteran lives in relation to where care exists. Assuming the Veteran is receiving care at the nearest facility, three means to reduce observed travel distance are (1) Veteran moves closer to care, (2) new care locations are opened, or (3) implementation of telehealth strategies. The geographic distribution of Veterans is mediated by external forces; however, the observed distance can help quantify shifts in travel burden as populations migrate around relatively fixed infrastructure. System level analysis of observed distances could allow for targeted interventions to address the latter two, through increased in-person resources or reinforced telehealth infrastructure.

A significant number of Veterans receive care at a VA facility that is further from their home than another VA facility offering the service. These Veterans typically travel distances 4–5 times greater than Veterans who receive care nearest home. The extra distance alone is almost twice that of the entire distance traveled by Veterans receiving care close to home. Excess travel distance provides utility as a facility-level measure, allowing for identification of facilities where Veterans could receive care, but are not.

Facility-level analysis can be performed by aggregating Veterans, and associated distances, by both the facility rendering care and the nearest facility. For many, these facilities are the same, but for a significant number they differ. In this manner, we can identify facilities “generating” excess travel due to Veterans within their catchment area seeking care elsewhere. Similarly, facilities “absorbing” excess travel by caring for Veterans outside their catchment area can be identified. These values are independent of community care referrals. Network maps can be generated from these relationships and lead to analysis of network efficiency, optimization, and exploration of causes. A table of the top facilities that generate/absorb excess travel is presented in Appendix 4. A detailed exploration of this topic is beyond the scope of this article.

The use of TeleSleep has generally been increasing and led to a significant reduction in travel. The median-avoided travel distance of TeleSleep is greater than the in-person equivalent’s observed travel distance, indicating TeleSleep initiatives may be reaching out farther into the community to help Veterans that otherwise may not receive sleep care. Although not all clinical encounters can be replaced by telehealth, for many situations it is a reasonable alternative to in-person care. Home-based methods can also be used as screening tools prior to scheduling in-person visits for rural Veterans who experience the greatest travel burden. Further expansion of telehealth services will have a significant reduction in Veteran travel, and increased reach into rural communities that otherwise would have limited access. Analysis of potential avoided travel distances through telehealth expansion in a region allows for targeted infrastructure development.

Our approach has several limitations. We utilized highly accurate geocoordinates; however, other commonly available geodata such as zip code could be used to generate similar results. Our measurements of travel distance do not represent the actual travel distance a Veteran would experience along road networks but provides a reasonable approximation. We know that travel burden is a complex concept that is not entirely explained by travel distance, nor does distance impact everyone equally. This area requires further research and development. Veterans may seek care further from home for a variety of reasons that cannot be fully explained here. We assumed that all telehealth visits occurred at home, and all HSAT devices were mailed to the Veteran; however, we know that some Veterans are required to travel to nearby clinics for telehealth visits or to receive HSAT devices. We believe that options promoting telehealth in the Veteran’s home represent best practices, and lead to significant reductions in travel. Thus, these calculations represent a reasonable approximation of the real-world Veteran experience.

The changes in healthcare delivery trends during this period are complex, and an exploration of the causes is beyond the scope of this article. Although COVID-19 had an impact, increased community referral via the MISSION act, and more rapid “re-opening” of community care also contributed. Our goal is to offer observed, excess, and avoided distance measures as tools to better quantify the impact of healthcare changes, rather than to explain the underlying etiology. The long-term impact of the pandemic and increased community care access on healthcare utilization will require further research.

## CONCLUSION

Observed and excess travel distance are valuable measures to quantify a major healthcare access barrier experienced by Veterans, and especially by rural Veterans. These measures allow for assessment of novel healthcare approaches to improve Veteran healthcare access (e.g., the VA TeleSleep initiative), and identification of specific regions that may benefit from additional in-person resources.

## Supplementary Information

Below is the link to the electronic supplementary material.

Supplementary file1 (PDF 657 KB)

## Data Availability

The datasets analyzed during the current study available from the corresponding author on reasonable request.

## Declarations:

### Disclaimer:

The contents of this article do not represent the views of the U.S. Department of Veterans Affairs or the United States Government.
