# Supplementary material for: Travel Burden as a Measure of Healthcare Access and the Impact of Telehealth within the Veterans Health Administration
Source: J Gen Intern Med. 2023 Jun 20;38(Suppl 3):805–13. doi: 10.1007/s11606-023-08125-3 (PMC10356728; doi:10.1007/s11606-023-08125-3)
Supplement: Supplementary file 1 — Supplementary file1 (PDF 657 KB) [file 11606_2023_8125_MOESM1_ESM.pdf]

Appendix 1

Stop Codes

Stop Codes (formerly known as Decision Support System Identifiers) are used by VA medical facilities to monitor workload for all outpatient encounters and inpatient professional services. Outpatient workloads are tracked by primary stop codes and secondary stop codes. The following combinations were utilized to identify specific clinic types:

Sleep Medicine Provider

- 349 with no secondary stop code
- 349 with any of the following secondary stop codes: 185, 186, 188, 510

Virtual Sleep Provider—primary: 349; secondary: 690

CPAP Clinic Visit—primary: 349; secondary: 116

PSG—primary: 143; secondary: none

Current Procedural Terminology (CPT) Codes:

PSG: 95807, 95810, 95811

HSAT: 95800, 95801, 95806, G0398, G0399, G0400

Appendix 2

City Block distance is so named as it represents the path taken between two points when constrained to a grid of city streets, where straight-line travel along the diagonal is not permitted. The Approximated Euclidean Distance (AED) assumes that the Earth can be viewed as a flat surface over short distances and removes the complexities of calculating distances over spherical objects. In our use case, the AED has an acceptable level of accuracy compared to other more computationally intense methods.

Latitudinal distance

$$D_{LAT} = |69.09 \times (LAT_1 - LAT_2)|$$

Longitudinal distance

$$D_{LONG} = \left|69.09 \times (LONG_1 - LONG_2) \times \cos\left(\frac{LAT_C \times \pi}{180}\right)\right|$$

Mean latitude correction factor:

$$LAT_C = \overline{LAT} = \frac{LAT_1 + LAT_2}{2}$$

Total distance:

$$AED = D_{TOTAL} = \sqrt{(D_{LAT}^2 + D_{LONG}^2)}$$

Appendix 3

Appendix 4

Facilities generating excessive travel by not treating patients within their catchment area					
Station	Excess distance	Miles per patient	Sent count	Near count	Percent sent
K	53,509	67	796	287	73.5
L	30,097	66	454	954	32.2
M	15,183	85	178	1,115	13.8
N	14,309	148	97	657	12.9
O	13,781	46	297	1,647	15.3
P	10,978	43	258	1,490	14.8
Q	10,343	89	116	35	76.8
R	10,269	66	156	1,145	12.0
S	7886	55	143	330	30.2
T	7361	102	72	444	14.0

Only in-person office visits to a sleep provider in 2021 are shown. Excess travel distances can be evaluated at the facility level to determine which facilities are generating a disproportionate amount of excess travel by not treating Veterans within their catchment area. “Excess distance”

is the total number of excess miles that were traveled by all Veterans who could have received care at the facility but went elsewhere. “Miles per patient” is the mean distance traveled per patient seen at the facility. “Sent count” is the number of Veterans who could have been treated at that facility but were treated at another facility that was further away from home. “Near count” is the number of Veterans treated at the facility and for whom the facility was the closest facility offering the medical service. “Percent sent” is the percentage of the potential patient base that received care at distant facilities. All stations have been de-identified

Facilities treating a disproportionate number of Veterans from outside their catchment area					
Station	Excess distance	Miles per patient	Received count	Near count	Percent received
A	68,327	73	939	6009	13.5
B	22,653	65	348	429	44.8
C	21,973	73	300	1766	14.5
D	16,250	91	179	1086	14.2
E	14,651	48	308	1483	17.2
F	12,643	47	267	1654	13.9
G	12,341	70	176	970	15.4
H	12,256	77	159	2955	5.1
I	11,221	68	165	3,091	5.1
J	8743	64	137	605	18.5

Only in-person office visits to a sleep provider in 2021 are shown. Excess travel distance can be evaluated at the facility level to determine which facilities are managing a disproportionate number of Veterans from outside their catchment area. “Excess distance” is the total number of excess miles that were traveled by all Veterans who received care at the facility, while “Miles per patient” is the mean distance traveled per patient. “Received count” is the number of Veterans who were treated at each facility but could have been treated at another facility closer

to home. “Optimal near count” is the number of Veterans treated at the facility and for whom the facility was the closest facility offering the medical service. “Percent received” is the percentage of the patient base that were treated at the facility but could have been treated closer to home. All stations have been de-identified
